# A simple theoretical framework for understanding heterogeneous differentiation of CD4^+^ T cells

**DOI:** 10.1186/1752-0509-6-66

**Published:** 2012-06-14

**Authors:** Tian Hong, Jianhua Xing, Liwu Li, John J Tyson

**Affiliations:** 1 Genetics, Bioinformatics, and Computational Biology Program, Virginia Polytechnic Institute and State University, Blacksburg, VA, 24061, USA; 2 Department of Biological Sciences, Virginia Polytechnic Institute and State University, Blacksburg, VA, 24061, USA

## Abstract

**Background:**

CD4^+^ T cells have several subsets of functional phenotypes, which play critical yet diverse roles in the immune system. Pathogen-driven differentiation of these subsets of cells is often heterogeneous in terms of the induced phenotypic diversity. *In vitro* recapitulation of heterogeneous differentiation under homogeneous experimental conditions indicates some highly regulated mechanisms by which multiple phenotypes of CD4^+^ T cells can be generated from a single population of naïve CD4^+^ T cells. Therefore, conceptual understanding of induced heterogeneous differentiation will shed light on the mechanisms controlling the response of populations of CD4^+^ T cells under physiological conditions.

**Results:**

We present a simple theoretical framework to show how heterogeneous differentiation in a two-master-regulator paradigm can be governed by a signaling network motif common to all subsets of CD4^+^ T cells. With this motif, a population of naïve CD4^+^ T cells can integrate the signals from their environment to generate a functionally diverse population with robust commitment of individual cells. Notably, two positive feedback loops in this network motif govern three bistable switches, which in turn, give rise to three types of heterogeneous differentiated states, depending upon particular combinations of input signals. We provide three prototype models illustrating how to use this framework to explain experimental observations and make specific testable predictions.

**Conclusions:**

The process in which several types of T helper cells are generated simultaneously to mount complex immune responses upon pathogenic challenges can be highly regulated, and a simple signaling network motif can be responsible for generating all possible types of heterogeneous populations with respect to a pair of master regulators controlling CD4^+^ T cell differentiation. The framework provides a mathematical basis for understanding the decision-making mechanisms of CD4^+^ T cells, and it can be helpful for interpreting experimental results. Mathematical models based on the framework make specific testable predictions that may improve our understanding of this differentiation system.

## Background

CD4^+^ T helper cells serve as key players in host immune responses by regulating and coordinating a large repertoire of immune cells, such as macrophages, B cells and CD8^+^ T cells. Consequently, CD4^+^ T helper cells are critical in human health ranging from homeostasis to pathogenesis of diseases [1,2]. Central to the functions of CD4^+^ T cells is their ability to produce a wide range of extracellular immunomodulating agents including cytokines and chemokines [3]. In order to correctly direct the immune response to antigen stimulation, CD4^+^ T cells have to secrete appropriate types of cytokines in appropriate amounts, and they achieve this by differentiating into various subtypes of functional CD4^+^ T cells from a pool of precursor cells, known as naïve CD4^+^ T cells. These subsets primarily include T helper 1 (T_H_1), T helper 2 (T_H_2), T helper 17 (T_H_17) and induced regulatory T (iT_Reg_) cells. Each subtype of CD4^+^ T cells produces a distinctive spectrum of cytokines, and in each of these subtypes there is typically one key transcription factor, or master regulator, that is highly expressed and controls the expression of downstream genes, including those encoding the lineage specific cytokines. The master regulators for the four functional subsets are T-bet, GATA3, RORγt and Foxp3, respectively [3].

The differentiation of CD4^+^ T cells is a highly controlled process, and the lineage specificity of the differentiation process is determined by integrating micro-environmental cues that activate various signaling pathways. These pathways include the T cell receptor (TCR) pathway and the Signal Transducer and Activator of Transcription (STAT) pathways [4,5], which are activated by cognate antigens and cytokines, respectively. Other pathways, such as those associated with Notch and Toll-like receptors (TLRs), are also involved in differentiation of CD4^+^ T cells into distinct lineages [6-8].

In a few types of chronic infections, the dominance of one subtype of CD4^+^ T cells can be observed [9]. However, most immune responses elicit balanced phenotypes of functional CD4^+^ T cells and their effector molecules, suggesting the importance of maintaining the diversity and flexibility of functional CD4^+^ T cells [10,11]. The importance of balancing the phenotypic composition is further corroborated by the fact that inappropriate dominance of particular subtype(s) of CD4^+^ T cells is often associated with inflammatory disorders [12-14]. It is not surprising to observe the balanced phenotypes of CD4^+^ T cells *in vivo*, given the plausible heterogeneous micro-environments of the naïve CD4^+^ T cells, which may stimulate the differentiation into multiple subtypes of functional CD4^+^ T cells. Interestingly, however, highly purified naïve CD4^+^ T cells can be induced to differentiate into multiple subtypes simultaneously in certain homogeneous *in vitro* experimental conditions [15-21]. Also interesting are the observations that optimum experimental conditions for generating homogeneous subsets of CD4^+^ T cells often include conditions that block the differentiation of undesired subsets [3]. These observations suggest that some highly regulated mechanisms, intrinsic to naïve CD4^+^ T cells, generate and maintain phenotypic heterogeneity of functional CD4^+^ T cells. *In vitro* assays showing heterogeneous differentiation recapitulate, at least in part, the balanced CD4^+^ T cell populations observed *in vivo*. Understanding situations of induced heterogeneous differentiation will shed light on the mechanisms controlling the response of populations of CD4^+^ T cells under physiological conditions.

Although the overexpression of one type of master regulator is generally considered the hallmark of the differentiation of one subtype of CD4^+^ T cells, it has been recently discovered that cells highly expressing two types of master regulators exist *in vivo*[16,17,22-26], and some of these 'double-positive' phenotypes have been shown to be important in responding to pathogens [16,17,26]. Consistent with *in vivo* studies showing that the numbers of single-positive and double-positive CD4^+^ T cells can be increased in comparable proportions upon pathogenic challenges [16], *in vitro* induction of the differentiation of double-positive CD4^+^ T cells often requires heterogeneous differentiation, which is accompanied by the differentiation of single-positive phenotypes [15-17]. Some double-positive CD4^+^ T cells can be generated by reprogramming the single-positive phenotypes, which also results in a heterogeneous population containing both single-positive and double-positive cells [23,24]. These experiments provide us with the clues to the conditions for generating double-positive phenotypes and highlight the intimate link between the double-positive phenotype and heterogeneous differentiation.

**Figure 1:**
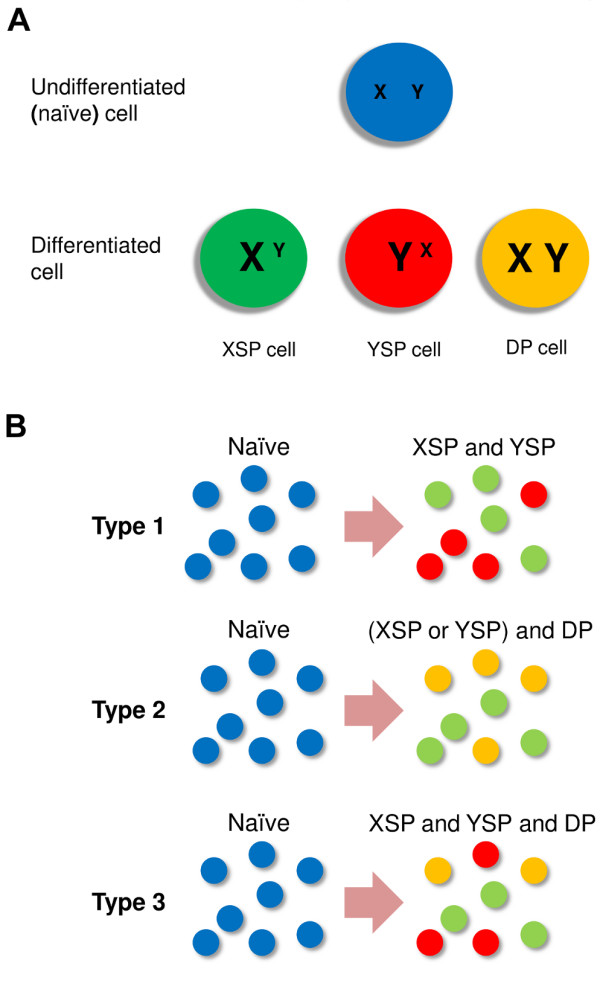
**Induced heterogeneous differentiation of CD4**^**+**^**T cells with respect to a pair of master regulators (X and Y). a.** Diversity of cell phenotypes during induced differentiation. In the undifferentiated cell, the expression level of both X and Y are low. When the cell is differentiated, three possible functional phenotypes can be obtained: X single-positive cell, Y single-positive cell and double-positive cell. **b.** Three types of induced heterogeneous differentiation. In a differentiation event, a group of naive cells can be differentiated into two types of single-positive cells (Type 1), one type of single-positive cell and DP cell (Type 2) or all three functional phenotypes (Type 3).

In most experiments demonstrating induction of heterogeneous differentiation, the expression levels of master regulators controlling two population subsets are examined at the single cell level. Despite the limited scope of these experiments in terms of the number of subsets considered, significant diversity of heterogeneous differentiation has been revealed. In a particular differentiation event, one can obtain one of the following types of heterogeneous populations (Figure 1): a population containing two types of single-positive cells [18], a population containing one type of single-positive cells and double-positive cells [17], and a population containing two types of single-positive cells and double-positive cells [15]. The diversity of heterogeneous differentiation in this minimum paradigm might be only the tip of an iceberg of complexity involving heterogeneous differentiation of all subsets of CD4^+^ T cells, but understanding a minimal system with only two classical subtypes is surely the place to start.

Previously, mathematical modeling has advanced our understanding of CD4^+^ T cell differentiation [27-32]. In particular, Höfer et al. [27] used a mathematical model to explain T_H_2 cell fate memory created by positive feedbacks in the signaling network; Mariani et al. [28] used a similar model to demonstrate the robust lineage choice between T_H_1 and T_H_2 cells; Yates et al. [29] linked the dynamics of master regulators to the phenotypic composition of T_H_1 and T_H_2 cells during differentiation and reprogramming; van den Ham et al. [30] used a generic model to describe the switches among all CD4^+^ T cell lineages; and Naldi et al. [32] developed a Boolean-network model that takes all four lineages of CD4^+^ T cells into consideration. We recently used a mathematical model to study the reciprocal differentiation of T_H_17 and iT_Reg_ cells, in which heterogeneous differentiation is observed [33]. It is unclear, however, how a broader spectrum of CD4^+^ T cells can be involved in heterogeneous differentiation and what determines the observed types of differentiated states.

Here, we propose a simple theoretical framework for understanding the heterogeneous differentiation of CD4^+^ T cells. We analyze the dynamic properties of a signaling network motif common to all CD4^+^ T cell lineages. We show that, at the level of cell populations, this motif can generate all possible homogeneous and heterogeneous phenotypic compositions with respect to a pair of master regulators, and at the single-cell level it ensures the robust commitment of a particular choice of differentiated state. Two types of positive feedback loops in this network motif govern three types of bistable switches, which in turn, result in three types of heterogeneous differentiation upon receiving appropriate combinations of input signals. This framework facilitates not only an intuitive understanding of the complex process by which CD4^+^ T cells integrate multiple signals to give rise to multiple functional phenotypes, but also the construction of more detailed mathematical models for studying CD4^+^ T cell differentiation. We provide three prototype models illustrating how to use this framework to explain experimental observations and make specific testable predictions.

## Results and discussion

### A basal signaling network motif is proposed to govern the differentiation of all lineages of CD4^+^ T cells

To consider the heterogeneous differentiation of CD4^+^ T cells, we introduce a minimal model based on a pair of master regulators (proteins X and Y). We neglect the influence of other master regulators during the differentiation process. In the undifferentiated (naïve) cell, the expression levels of X and Y are both low, and the stable expression of either X or Y marks the differentiation event. Three phenotypes can be observed upon differentiation: X single-positive (XSP) cell, Y single-positive (YSP) cell, and double-positive (DP) cell (Figure 1A). In the model, heterogeneous differentiation is defined as the process in which more than one functional (non-naïve) phenotypes can be observed upon uniform treatment of a population of simulated naïve cells (see Methods).

In this minimum paradigm, three types of heterogeneous differentiation can be induced: 1) two different types of single-positive cells are differentiated simultaneously from naïve precursors; 2) one type of single-positive cells differentiates simultaneously with double-positive cells; and 3) both types of single-positive cells differentiate simultaneously with double positive cells (Figure 1B). We define these three scenarios as Type 1, 2 and 3 heterogeneous differentiations, respectively.

**Figure 2:**
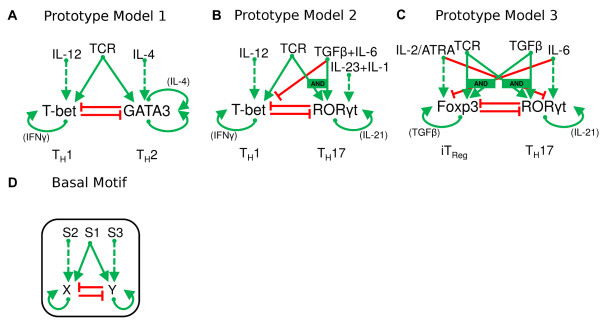
**Basal network motif controlling heterogeneous differentiation in the two master regulator paradigm.** Solid green arrow: activation influence in which the activator alone can switch on the expression of the target protein. Dashed green arrow: activation influence in which the activator alone cannot switch on the expression of the target protein. Red arrow: inhibition influence. Protein name in parenthesis: possible intermediate protein for the positive feedback loop. **a.** Prototype Model 1: heterogeneous differentiation of T_H_1 and T_H_2. **b.** Prototype Model 2: heterogeneous differentiation of T_H_1 and T_H_17. **c.** Prototype Model 3: heterogeneous differentiation of iT_Reg_ and T_H_17. **d.** The basal network motif.

We next propose a basal network motif that governs cell differentiation in this minimal model. Based on known molecular interactions, we notice that the four master regulators of CD4^+^ T cells are all involved in signaling networks of similar topologies (Figure 2A-C). From these examples, we introduce a ‘basal motif’ (Figure 2D). In the basal motif, two master regulators (X and Y) mutually inhibit each other’s expression, while activating their own production. Two types of signals are responsible for activating the expression of the master regulators: a 'primary signal' (S1) which is sufficient to fully upregulate at least one master regulator, and two polarizing signals (S2 and S3) which favor the expression of one master regulator or the other (X and Y, respectively) but are not sufficient to upregulate their expression in the absence of a primary signal (Figure 2D). Each influence relationship in this basal motif has direct biological meaning, but some components in this motif may represent different biological entities in different dual-master-regulator networks. For example, in the T_H_1-T_H_2 network (Figure 2B) the primary signal represents the TCR ligands, whereas in the iT_Reg_-T_H_17 network (Figure 2C) it represents a combined treatment of TCR ligands and TGFβ, which is justified by the fact that both TCR and TGF-β signaling pathways activate both Foxp3 and RORγt. Note that the signals, which are treated as parameters in our models, represent exogenous cytokine doses only, not endogenous cytokines produced by T cells upon activation. The latter are represented in part by the auto-activation relations.

In Table 1, we list the generic signaling components and their corresponding biological entities for each prototype model. Note that a TCR ligand is a typical example of a primary signal, and certain groups of cytokines correspond to polarizing signals. In Table 2, we list the evidences for all molecular influences of each prototype model.

**Table 1:** Signaling components in basal motif and their corresponding biological components in prototype models

**Model**	**Generic signaling component**	**Corresponding biological component**
Prototype 1	Primary signal (S1)	TCR signal
Prototype 1	Polarizing signal 1 (S2)	Exogenous IL-12
Prototype 1	Polarizing signal 2 (S3)	Exogenous IL-4
Prototype 1	Master regulator 1 (X)	T-bet
Prototype 1	Master regulator 2 (Y)	GATA3
Prototype 2	Primary signal (S1)	TCR signal
Prototype 2	Polarizing signal 1 (S3-1)	Exogenous IL-23 + IL-1 signal
Prototype 2	Polarizing signal 2 (S3-2)	Exogenous TGF-β + IL-6 signal
Prototype 2	Master regulator 1 (X)	T-bet
Prototype 2	Master regulator 2 (Y)	RORγt
Prototype 3	Primary signal (S1)	TCR + Exogenous TGF-β signal
Prototype 3	Polarizing signal 1 (S2)	Exogenous ATRA/IL-2 signal
Prototype 3	Polarizing signal 2 (S3)	Exogenous IL-6 signal
Prototype 3	Master regulator 1 (X)	Foxp3
Prototype 3	Master regulator 2 (Y)	RORγt

**Table 2:** Evidences for molecular influences in prototype models

**Model**	**Molecular Influence**	**Evidence**
Prototype 1	TCR signal upregulates T-bet expression	[34]
Prototype 1	TCR signal upregulates GATA3 expression	[35]
Prototype 1	IL-12 signal upregulates T-bet expressionin the presence of TCR signal	[34]
Prototype 1	IL-4 signal upregulates GATA3 expressionin the presence of TCR signal	[18,36]
Prototype 1	T-bet inhibits GATA3 expression	[37]
Prototype 1	GATA3 inhibits T-bet expression	[38]
Prototype 1	T-bet promotes its own expression	[39]
Prototype 1	GATA3 promotes its own expression	[40]
Prototype 2	TCR signal upregulates T-bet expression	[34]
Prototype 2	TCR signal upregulates RORγt expression in the presence of TGF-β	[41,42]
Prototype 2	IL-23 + IL-1 signal upregulates RORγt expressionin the presence of TCR signal	[17]
Prototype 2	TGF-β signal upregulates RORγt expressionin the presence of TCR signal	[17]
Prototype 2	TGF-β signal downregulates T-bet expression	[43]
Prototype 2	T-bet inhibits RORγt expression	[44]
Prototype 2	RORγt inhibits T-bet expression	[45]
Prototype 2	T-bet promotes its own expression	[39]
Prototype 2	RORγt promotes its own expression	[11,46]
Prototype 3	TCR signal upregulates Foxp3 expressionin the presence of TGF-β	[41,42]
Prototype 3	TCR signal upregulates RORγt expressionin the presence of TGF-β	[41,42]
Prototype 3	TGF-β signal upregulates Foxp3 expressionin the presence of TCR signal	[41,42]
Prototype 3	TGF-β signal upregulates RORγt expressionin the presence of TCR signal	[41,42]
Prototype 3	IL-6 upregulates RORγt expression	[47]
Prototype 3	IL-6 downregulates Foxp3 expression	[47]
Prototype 3	ATRA/IL-2 upregulates Foxp3 expression	[48,49]
Prototype 3	ATRA/IL-2 downregulates RORγt expression	[48,49]
Prototype 3	Foxp3 inhibits RORγt expression	[50]
Prototype 3	RORγt inhibits Foxp3 expression	[51]
Prototype 3	Foxp3 promotes its own expression	[11]
Prototype 3	RORγt promotes its own expression	[11,46]

We first analyze Type 1 heterogeneous differentiation using the core motif, in the absence of auto-activation, and then we use the full version of the basal motif to explain all three types of heterogeneous differentiation.

### The basal motif without auto-activations can generate Type 1 heterogeneous differentiation

#### The symmetric case

**Figure 3:**
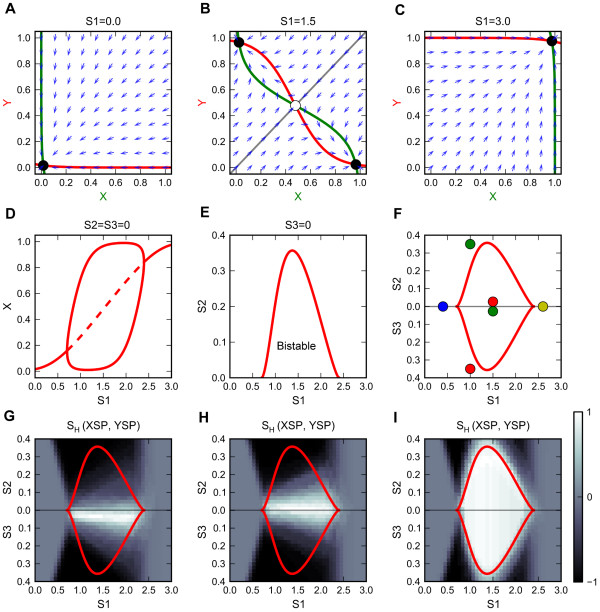
**Analyses of the core motif with symmetrical parameters. a-c.** Phase plane portraits for three values of primary signal strength (zero, intermediate, high), in the absence of polarizing signals (S2 = S3 = 0). Green curve: X nullcline; red curve: Y nullcline; blue arrow: representative vector in the phase space; closed circle: stable steady state; open circle: unstable steady state; gray curve: separatrix. **d.** One-parameter bifurcation diagram for steady state level of X as a function of primary signal S1. Solid curve: stable steady state; dashed curve: unstable steady state. **e.** Two-parameter bifurcation diagram with respect to primary signal S1 and polarizing signal S2, with S3 = 0. Solid curve: locus of pitchfork bifurcation points. The pitchfork bifurcation points coalesce and disappear at S2 = 0.357. **f.** Bidirectional two-parameter bifurcation diagram with respect to primary signal S1 and polarizing signals S2 and S3. Top half: S1—S2 diagram, with S3 = 0, as in panel E. Bottom half: S1—S3 diagram, with S2 = 0. The types of stable steady states in each region are annotated as colored circles. Adjoined circles: multistability. See Figure 1 for interpretation of the color scheme. **g.** Simulation results for treatment of a population of cells simultaneously with primary and polarizing signals. **h.** Simulation results for sequential treatment: polarizing signal followed by primary signal. **I.** Simulation results for sequential treatment: primary signal followed by polarizing signal. In G-I, the heterogeneity scores with respect to XSP and YSP are plotted.

Consider first the case of perfectly symmetrical parameter settings (Additional file 1: Table S1 Generic Model 1) for the core motif without self-activations. (See the Methods section for a description of our mathematical model of the signaling motifs.) In the absence of exogenous signals, the system persists in the stable ‘double-negative’ state corresponding to naïve cells (Figure 3A). Small positive values of the primary signal (0 < S1 < 0.704) drive the expression of modest amounts of both master regulators in a single cell. Larger values (0.704 < S1 < 2.396) destabilize the co-expression state and give rise to two new (alternative) stable steady states: the X-high-Y-low state and the X-low-Y-high state, which correspond to XSP and YSP cells, respectively (Figure 3B). The basins of attraction of these two states are separated by the diagonal line (*X* = *Y*) through the state space. When the primary signal is extremely strong (S1 > 2.396), the system is attracted to a unique stable steady state (X-high-Y-high), corresponding to a DP cell (Figure 3C). Bifurcation analysis on these steady states shows that the system undergoes pitchfork bifurcations at S1 = 0.704 and at S1 = 2.396 (Figure 3D), a typical type of bifurcation obtained for dynamical systems with perfect symmetry [52-54]. Saturation of the primary signal may prevent cells from reaching the DP state (Additional file 2: Figure S1A and B).

The presence of a polarizing signal breaks the symmetry of the system, resulting in a pitchfork bifurcation with broken symmetry (Additional file 3: Figure S2A and B). To analyze the influence of polarizing signals on this dynamical system, we plot two-parameter bifurcation diagrams with respect to the primary signal and to each of the polarizing signals (e.g., Figure 3E, for S1 and S2). In Figure 3F we plot a ‘bidirectional’ two-parameter bifurcation diagram, with S2 versus S1 plotted ‘up’ and S3 versus S1 plotted ‘down’ (see Methods for details). In Figure 3F we see a bistable region (bounded by the red curves) for moderate values of the primary signal strength (0.7-2.3 units) and for low values (0–0.35 units) of either of the polarizing signal strengths. Within the bistable region are found the two types of single-positive states. Outside the bistable region are found unique steady state solutions that vary continuously from the naïve state on the left to the double-positive state on the right, through intermediate region (0.7 < S1 < 2.3) dominated by XSP cells (for S2 > 0) or by YSP cells (for S3 > 0). Because of the perfect symmetry of the parameters, both of the cusps of the bistable region lie on the X-axis.

In order to predict the response of this regulatory system to changing stimuli (S1 and S2, or S1 and S3), we must be careful in interpreting the effects of trajectories crossing the two-parameter bifurcation diagram in Figure 3F. If we fix the polarizing signals at S3 = 0, S2 = 0.1 and increase the primary signal from 0 to 3, as in Additional file 3: Figure S2A and B, we see that the regulatory system passes smoothly from the naïve state (X-low-Y-low) to the XSP state (X-high-Y-low) to the DP state (X-high-Y-high). The regulatory system passes over the bistable region without undergoing any abrupt changes of the state (bifurcation) or exhibiting hysteresis effects. On the other hand, if we fix the primary signal at S1 = 1.5 and increase one of the polarizing signals (either S2 or S3), as in Additional file 3: Figure S2 C and D, we see that the regulatory system starts in one of the single-positive state and jumps abruptly to another single-positive state at a saddle-node bifurcation point. Also, the system exhibit hysteresis because, if the polarizing signal is reduced to zero after the jump occurs, the regulatory system remains stuck in the stable ‘flipped’ state (XSP if S2 increases/decreases, YSP if S3 increases/decreases). We call this type of response a ‘reprogramming’ switch, because the control system flips irreversibly between alternative single-positive states. On the contrary, transitions from the naïve or the DP state to either one of the single-positive states are smooth and reversible (they do not invoke reprogramming).

We next show that this network motif can generate heterogeneous differentiation and identify the parameter region in which a heterogeneous population can be obtained. To this end we simulate the induced differentiation process in a group of cells (with small cell-to-cell variability) exposed to various combinations of primary (S1) and polarizing signals (either S2 or S3). For each combination of S1 and S2 (or S3), we compute the percentages of cells of different phenotypes in the final (steady state) differentiated population. We plot these percentages (as heat maps) over the coordinates of the bidirectional two-parameter bifurcation diagram (see Additional file 4: Figure S3A-D). We summarize these results with a ‘heterogeneity score’ (see Methods) to highlight the region of parameter space that can generate heterogeneous populations (Figure 3G). Not surprisingly, in the absence of strong polarizing signals (S2 ≈ 0 and S3 ≈ 0), the primary signal can induce heterogeneous differentiation of two single-positive phenotypes (Figure 3G, bright area). This is because of the close proximity of the naïve states to the separatrix, and the presence of cell-to-cell variability which can bias individual cells towards different phenotypes (Additional file 4: Figure S3E). The polarizing signal, on the other hand, makes the differentiation into one single-positive phenotype more likely, which can result in homogeneous differentiation once it is sufficiently strong (Figure 3G, dark area).

We next explore how the cell population responds to sequential stimuli rather than simultaneous stimuli. If the population is stimulated first by a polarizing signal and then, after the cells have reached their steady states, the simulations are continued in the presence of primary signal, we find that the response to sequential stimuli is very similar to the response to simultaneous stimuli (Figure 3H). But when we switch the sequence of the stimuli, the polarizing signal fails to influence cell fate in the bistable region, resulting in heterogeneous populations in this region (Figure 3I). This is due to a hysteresis effect, which prevents reprogramming by polarizing signals that are insufficiently strong. These results suggest that polarizing signals can influence cell fate determination until the induction of differentiation, after which their influence is greatly reduced.

#### Broken symmetry

**Figure 4:**
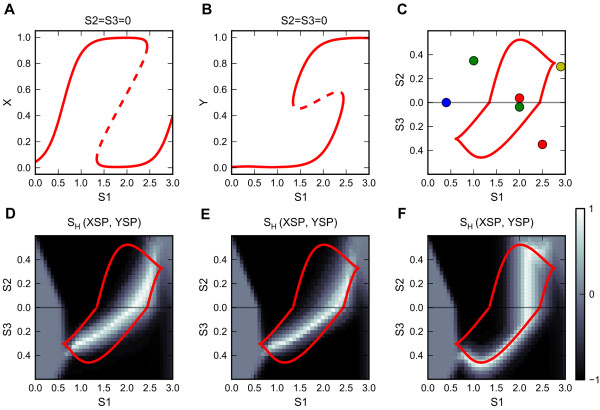
**Analyses of the core motif with asymmetrical parameters. a and b.** One-parameter bifurcation diagram for steady state levels of X and Y as functions of primary signal S1 (S2 = S3 = 0). **c.** Bidirectional two-parameter bifurcation diagram with respect to primary signal S1 and polarizing signal S2 or S3. See legend of Figure 3 panels D and E for the interpretation of curves and colored circles. **d-f.** See legend of Figure 3 Panels G-I for simulation conditions.

The preceding analysis is based on a set of perfectly symmetrical parameters in the signaling network, although the exogenous polarizing signals can act as ‘symmetry breakers’. How differently does the regulatory system behave if its intrinsic kinetic parameters are not perfectly symmetrical? For illustrative purposes, we use a representative set of asymmetrical parameter values (Additional file 1: Table S1 Generic Model 2). Because of the asymmetries, the primary signal upregulates the two master regulators at different thresholds (Figure 4A and B), and the bistable region of the bidirectional two-parameter bifurcation diagram is re-oriented so that its cusps are located on different sides of the X-axis (Figure 4C). When we stimulate cell populations with combinations of primary and polarizing signals, we find that the parameter region that gives rise to heterogeneous populations is not coincident with the X-axis. Instead, the ‘heterogeneous’ region forms a patch that intersects the X-axis (Figure 4D). In this situation, the system requires a specific range of primary signal strength to generate a heterogeneous population. On the other hand, the primary signal now gains some control over cell fate determination, in addition to its ability to trigger the differentiation. For a similar network in B cells, Sciammas et al. [55] recently showed that the strength of the B cell receptor signal (primary signal) can determine cell fate because of the asymmetry of the network.

The effects of sequential stimuli in the asymmetrical model are similar to their effects in the symmetrical model (Figure 4E and F).

Up to this point, we have assumed that the relaxation rates of X and Y are identical $\left(\gamma_{\text{X}}=\gamma_{\text{Y}}=5\right)$. Breaking this symmetry changes the parameter combinations that generate heterogeneous differentiation without changing the bifurcation diagram (Additional file 5: Figure S4). This result, together with the responses to sequential stimuli discussed earlier, shows that although the bistable region is critical to obtaining heterogeneous differentiation, the exact phenotypic composition within the bistable region also depends on the kinetics of the signal inputs and the intrinsic relaxation rates of the master regulators.

We suggest that biological signaling networks of this type (i.e., those resembling the basal motif) may have evolved to take advantage of either symmetrical or asymmetrical types of behavior. A typical asymmetrical design is found in the T_H_1 and T_H_2 paradigm, in which TCR signaling not only triggers the heterogeneous differentiation of both T_H_1 and T_H_2, but also regulates their phenotypic compositions depending on signal strength (discussed in detail in later section). With this understanding, one can design experiments to study more detailed signal-control principles of a particular signaling network governing heterogeneous differentiation.

### The basal network motif with additional positive feedback loops can generate all types of heterogeneous differentiation

Previously, mathematical modelers found that interconnected positive feedback loops can give rise to complex multistability in CD4^+^ T cell differentiation [28] and elsewhere [54]. It is still not clear, however, how these different multistable regions depend on the interconnection of multiple positive feedback loops, nor how one can use biologically relevant signals to guide cells into various multistable regions, where heterogeneous differentiation might occur. In this section, we show that our basal motif can give rise to complex multistability, we clarify the effects of the additional positive feedback loops using bifurcation analysis, and we explain the biological meaning of each parameter region in the context of the heterogeneous differentiation of CD4^+^ T cells.

**Figure 5:**
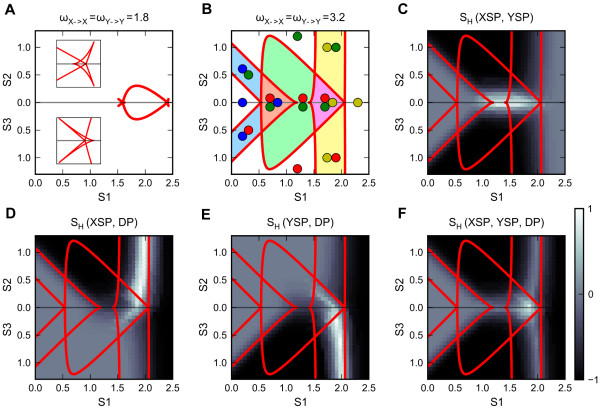
**Analyses of the basal motif with auto-activation relations. a.** Bidirectional two-parameter bifurcation diagram with respect to primary signal S1 and polarizing signals S2 and S3 for intermediate weight of auto-activation relations (ω = 1.8). Insets show the zoomed-in view of the cusp regions of the bistable region. **b.** Bidirectional two-parameter bifurcation diagram with respect to primary signal S1 and polarizing signals S2 and S3 for high weight of auto-activation relations (ω = 3.2). The types of stable steady states in each region are annotated as colored circles. Adjoined circles: multistability. See Figure 1 for interpretation of the color scheme. Light blue area: bistable region governing differentiation switch. Light green area: bistable region governing reprogramming switch. Light yellow area: bistable region governing co-expression switch. **c-f.** Various types of heterogeneity scores are plotted for high weight of auto-activation relations (ω = 3.2). **c.** The heterogeneity scores with respect to XSP and YSP. **d.** The heterogeneity scores with respect to XSP and DP. **e.** The heterogeneity scores with respect to YSP and DP. **f.** The heterogeneity scores with respect to XSP, YSP and DP.

For illustrative purpose, we first choose another set of perfectly symmetrical parameters (Additional file 1: Table S1 Generic Model 3). This model differs from Generic Model 1 in that the double-negative feedback (mutual inhibition) is not strong enough to create bistability. Nonetheless, with the addition of symmetrical increase of auto-activation loops, a bistable region first appears in the intermediate range (1.7 < S1 < 2.4) of the primary signal (Additional file 6: Figure S5A), similar to the case of Generic Model 1 (Figure 3D). Further increase of the auto-activation weights enlarges the bistable region, and at a critical point (weights = 1.8), the pitchfork bifurcation changes from supercritical (Additional file 6: Figure S5A, weights = 1.5) to subcritical (Additional file 6: Figure S5B, weights = 3.2). Beyond the transition from supercritical to subcritical, each pitchfork bifurcation gives rise to two saddle-node bifurcation points (Additional file 6: Figure S5B and C). On the bidirectional (S1-S2-S3) two-parameter bifurcation diagram (Figure 5A), each cusp region 'folds back' to form three interconnected cusp regions, which govern two new bistable regions and one tristable region (Figure 5A). Further increase of the auto-activation weights enlarges the original bistable region as well as the newly formed multistable regions. Eventually, the plane on the bidirectional two-parameter bifurcation diagram is divided into 11 regions with distinct stability features (Figure 5B).

We clarify this unique two-parameter bifurcation diagram as follows. If the autoactivation loops are absent or weaker, the parameter region outside of the reprogramming switch bistable region (Figure 3F) is continuous and monostable, although it can represent four types of steady states. Essentially, strong auto-activation loops create folding in this monostable region so that it is divided into four monostable regions separated by four new bistable regions. This structure effectively creates an additional level of robustness of cell fate commitment, which is rendered by two new types of bistable switches, in addition to the reprogramming switch. One type of switch consists of the two bistable regions located at lower range of the primary signal (Figure 5B, light blue areas), which controls differentiation/dedifferentiation commitment, i.e. the switches from or to the naïve state (Additional file 6: Figure S5D and E). Another type of switch consists of the two bistable regions located at higher range of the primary signal (Figure 5B, light yellow areas), which controls co-expression commitment, i.e. the switches from or to the double-positive state (Additional file 6: Figure S5D and E). We define these two switches as the ‘differentiation switch’ and the ‘co-expression switch’ respectively. The tri-stable regions in this diagram are the overlapping areas between the bistable regions governed by the reprogramming switch and either the differentiation or the co-expression switch. In fact, extremely high weights (>4) of auto-activation may give rise to a tetra-stable region, where the three types of the bistable regions overlap (Additional file 6: Figure S5C).

In summary, the positive feedback loop involving mutual inhibition of the master regulators can create the reprogramming switch, and additional feedback loops involving auto-activation can enhance the robustness of the reprogramming switch and create the differentiation switch and the co-expression switch. The features of the three bistable switches are listed in Table 3.

**Table 3:** Features of three bistable switches obtained with the basal motif

**Bistable switch**	**Phenotypic transition controlled by the switch**	**Underlying positive feedback loops**	**Type of related heterogeneous differentiation**
Differentiation	Naïve ⇔ XSP or YSP	Auto-activation	NA
Reprogramming	XSP ⇔ YSP	Created by mutual inhibition and enhanced by auto-activation	Type 1
Co-expression	XSP or YSP ⇔ DP	Auto-activation	Type 2

We next ran simulations to check whether these regions of multistability are correlated to various types of heterogeneous differentiation. Our results show that Type 1 heterogeneous differentiation can be induced in the reprogramming switch region (Figure 5C) (this is consistent with the results obtained with the core motif), Type 2 heterogeneous differentiation can be induced in the co-expression bistable switch regions (Figure 5D and E), and Type 3 heterogeneous differentiation can be induced in the tri-stable region consisting of three functional (non-naïve) states (Figure 5F). These types of heterogeneous differentiations are all robust in terms of single cell commitment because the corresponding parameter regions admit a variety of stable steady states.

Positive feedback loops have long been recognized as mechanisms for biological switches [56-58]. We have demonstrated that two types of positive feedback in the CD4^+^ T cell differentiation network underlie three types of bistable switches that govern the transitions among different phenotypes of those T cells. In addition to ensuring the robust commitment, the multistability created by positive feedback loops may be used to generate phenotypic diversities of various types. In this context, the biological functions of the positive feedback loops are seen as more versatile than giving rise to simple on-or-off switches.

Our theoretical analysis of the basal regulatory motif (Figure 2D) started with symmetrical parameter values and then considered the effects of broken symmetries. In the next section, we show how non-symmetrical prototype models of heterogeneous differentiation among real lines of CD4^+^ T cells can be studied within this unifying framework despite their diverse features.

### Mathematical models based on the theoretical framework can be used to understand experimental results and make testable predictions

In this section we discuss three prototype models for studying heterogeneous differentiation of CD4^+^ T cells. The first two models are aimed to explain some interesting biological phenomena that were not studied previously with mathematical modeling. The third one is a simplified version of our previous model [33], but we have made it more accessible by using the framework presented here. Because of their limited scope, none of these models are intended to provide a comprehensive understanding of the corresponding biological systems. Rather, our intention is to illustrate how to use the modeling framework to explain observed heterogeneous differentiation and make testable predictions.

Prototype Model 1: Heterogeneous differentiation of T_H_1 and T_H_2 cells

Previous mathematical models successfully described the dynamic behavior and the underlying molecular control system of the reciprocal differentiation of T_H_1 and T_H_2 cells [27-31]. However, heterogeneous differentiation of T_H_1 and T_H_2 cells and its underlying molecular controls were not studied with these models. Yamashita et al. [18] discovered that the heterogeneous differentiation of T_H_1 and T_H_2 cells can be obtained with antigenic stimulations. Similar observations were obtained by Hosken et al. [20], and Messi et al. [21]. We have built a mathematical model, based on the influence diagram in Figure 2A, to describe heterogeneous differentiation of T_H_1 and T_H_2 cells. The parameter values for the model are listed in Additional file 1: Table S2.

**Figure 6:**
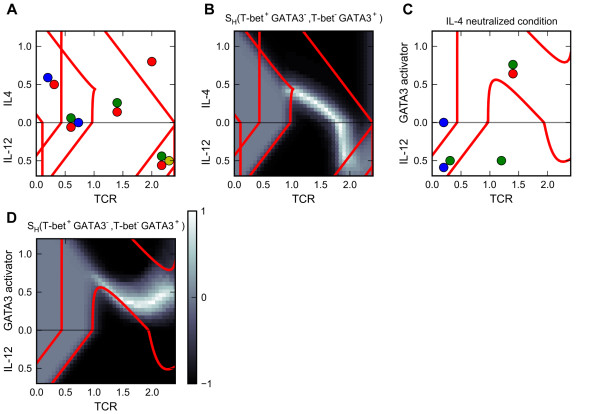
**Analyses of Prototype Model 1 (heterogeneous differentiation of T**_**H**_**1 and T**_**H**_**2 cells). a.** Bidirectional two-parameter bifurcation diagram with respect to primary signal TCR and polarizing signals IL-12 and IL-4. **b.** Simulation results for induced differentiation. The heterogeneity scores with respect to T-bet single-positive phenotype and GATA3 single-positive phenotype are shown. **c.** Same legend as Panel A. The GATA-3 auto-activation relation is blocked in the model. **d.** Same legend as Panel B. The GATA-3 auto-activation relation is blocked in the model. In Panels A and C: Adjoined circles: multistability. Blue circle: naïve phenotype. Green circle: T-bet single-positive phenotype. Red circle: GATA3 single positive phenotype. Yellow: DP phenotype.

Figure 6A shows the bidirectional two-parameter bifurcation diagram, and Figure 6B shows the simulation results as the heterogeneity score with respect to the two single-positive phenotypes. Our simulation results suggest that exogenous polarizing signals, i.e. IL-4 and IL-12, are not sufficient to trigger differentiation. They must be accompanied by a sufficiently high dose of antigenic stimulant (TCR signal) to trigger the differentiation into the corresponding phenotypes. This conclusion is in agreement with previous experimental results [18]. High strength of TCR signal alone (>1 unit) or with intermediate level of IL-4 (0.3 unit) was sufficient to induce the differentiation of two single-positive phenotypes. With increasing strengths of TCR signal, our simulations show a spectrum of heterogeneous populations with increasing percentages of T_H_2 cells and decreasing percentage of T_H_1 cells. The following experimental findings are consistent with our simulation. Messi et al. [21] observed the heterogeneous differentiation of T_H_1 and T_H_2 with IL-4 and antigenic stimulant. Yamashita et al. [18] observed a similar pattern of heterogeneous populations with increasing doses of antigenic stimulant in the presence of an intermediate level of IL-4. Hosken et al. [20] also observed such pattern with a different type of antigenic stimulant, although only a narrow range of stimulant concentrations could give rise to heterogeneous populations. Clearly, our model predicts that in order to achieve comparable proportions of T_H_1 cells and T_H_2 cells, one would need a higher dose of antigenic stimulant without exogenous IL-4 as compared to with exogenous IL-4. Based on the bifurcation diagram, we also predict that a slow increase of stimulant concentration favors the differentiation of T_H_1 cells. Additionally, the simulation results and bifurcation analysis show that the double-positive phenotype can be obtained in the presence of T_H_1 polarizing signals. Hegazy et al. [24] have discovered that exogenous T_H_1 polarizing signals can reprogram T_H_2 cells into T-bet^+^GATA3^+^ cells in the presence of antigenic stimulant. Our model predicts that the differentiation of such double-positive phenotype can be directly induced by high dose of antigenic stimulant (>2 units) in the presence of exogenous T_H_1 polarizing signals (0.5 unit), and the differentiation is likely to be heterogeneous with the concurrent induction of two types of single-positive cells, in addition to the double-positive cells. If we reduce the auto-activation weight of GATA3 (see Methods), then the TCR signal primarily triggers the differentiation of T_H_1 cells instead of a heterogeneous population (Figure 6C and D). Maruyama et al. [59] demonstrated that TCR signal alone can induce a significant fraction of GATA3^+^ cells (this is consistent with the experimental findings mentioned above), and blocking the auto-activation feedback between GATA3 and IL-4 prevents the induction of GATA3^+^ cells. Our model predicts that the population may be dominated by T_H_1 cells under this condition.

Table 4 summarizes the published observations consistent with our simulation results and new predictions based on the bifurcation analyses and simulation results.

**Table 4:** Summary of simulation results of Prototype Model 1

**Conditions of differentiation induction**	**Induced cell population**	**Evidence**
Exogenous polarizing signals alone	No induction of differentiation	[18]
Low dose of antigenic stimulant (TCR signal <1 units) and exogenous polarizing signals	Homogeneous differentiation (induced phenotype corresponds to type of polarizing signal)	[18]
Antigenic stimulant in the presence of IL-4	Heterogeneous differentiation of T_H_1 and T_H_2	[18,21]
Increasing strengths of TCR signal	A spectrum of heterogeneous populations with increasing percentages of T_H_2 cells and decreasing percentage of T_H_1 cells.	[20]
Increasing strengths of TCR signal in the presence of IL-4	A spectrum of heterogeneous populations with increasing percentages of T_H_2 cells and decreasing percentage of T_H_1 cells.	[18]
TCR signal alone vs. TCR signal with IL-4	Stronger TCR signal is required to achieve a balanced population of T_H_1 and T_H_2 in condition without IL-4 than in condition with IL-4	Prediction
TCR signal + T_H_1 polarizing signals	Double-positive phenotype can be observed (via reprogramming from T_H_2 cells)	[24]
TCR signal + T_H_1 polarizing signals	Direct induction of double-positive phenotype can be achieved with strong TCR signal and T_H_1 polarizing condition	Prediction
Blocking GATA3-IL4 feedback by antibodies against IL-4 and inducing with TCR signal	No T_H_2 cells are observed	[59]
Blocking GATA3-IL4 feedback by antibodies against IL-4 and inducing with TCR signal	Homogeneous differentiation of T_H_1 cells	Prediction

Prototype Model 2: Heterogeneous differentiation of T_H_1 and T_H_17 cells

**Figure 7:**
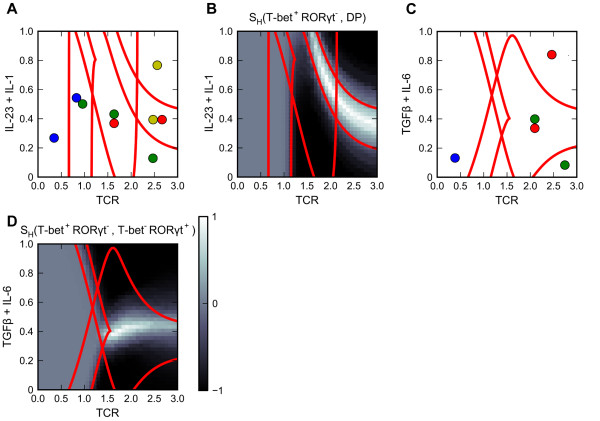
**Analyses of Prototype Model 2 (heterogeneous differentiation of T**_**H**_**1 and T**_**H**_**17 cells). a.** Two-parameter bifurcation diagram with respect to primary signal TCR and polarizing signal IL-23 + IL1. **b.** Simulation results for induced differentiation. The heterogeneity scores with respect to T-bet single-positive phenotype and DP phenotype are shown. **c.** Two-parameter bifurcation diagram with respect to primary signal TCR and polarizing signal TGF-β + IL-6. **d.** Simulation results for induced differentiation. Heterogeneity scores with respect to T-bet single-positive phenotype and RORγt single-positive phenotype are shown. In Panels A and C: Adjoined circles: multistability. Blue circle: naïve phenotype. Green circle: T-bet single-positive phenotype. Red circle: RORγt single-positive phenotype. Yellow: DP phenotype.

We build a prototype model to study the heterogeneous differentiation of T_H_1 and T_H_17 cells that was recently demonstrated by Ghoreschi et al. [17]. The influence diagram of the model is shown in Figure 2B, and the parameter values are listed in Additional file 1: Table S3. In the presence of TCR signal alone, the simulated population is dominated by T_H_1 cells (Figure 7A and B). When the TCR signal is combined with IL-23 + IL-1 polarizing signal, the induced population contains both the T-bet^+^RORγt^-^ single-positive phenotype and the T-bet^+^RORγt^+^ double positive phenotype (Figure 7A and B). When the TCR signal is combined with TGF-β (another polarizing signal), the population is dominated by the T-bet^-^RORγt^+^ single-positive phenotype (Figure 7C and D). These results are consistent with the observations of Ghoreschi et al. [17]. Our model predicts that lowering the TCR signal strength may result in the reprogramming from T-bet^+^RORγt^+^ double positive phenotype to T-bet^+^RORγt^-^ single positive phenotype even in the presence of a strong IL-23 + IL-1 signal and that when low dose of TGF-β + IL-6 (≈0.4 unit) is used, one may observe the heterogeneous differentiation of T_H_1 and T_H_17 cells. Also, the model recapitulates the scenario in which knocking out T-bet genes resulted in the homogeneous differentiation into T-bet^-^RORγt^+^ single-positive phenotype when either of the polarizing signals is used (Additional file 7: Figure S6) [17].

Simulation results with testable predictions are summarized in Table 5.

**Table 5:** Summary of simulation results of Prototype Model 2

**Conditions of differentiation induction**	**Induced cell population**	**Evidence**
TCR signal alone	The cell population is dominated by the T_H_1 cells	[17]
TCR signal and IL-23 + IL-1 signal	Heterogeneous differentiation of T-bet^+^RORγt^-^ cells and T-bet^+^RORγt^+^ cells.	[17]
TCR signal and TGF-β + IL-6 signal	The cell population is dominated by T-bet^-^RORγt^+^ cells	[17]
Lowering TCR signal after differentiation	Reprogramming from T-bet^+^RORγt^+^ cells to T-bet^+^RORγt^-^ cells	Prediction
TCR signal and low dose of TGF-β + IL-6 (≈0.4 unit)	Heterogeneous differentiation of T_H_1 and T_H_17 cells	Prediction
Knocking out T-bet genes and inducing with TCR signal	Homogeneous differentiation of T-bet^-^RORγt^+^ cells with either TGF-β signal or IL-23 + IL-1 signal	[17]

Prototype Model 3: Heterogeneous differentiation of iT_Reg_ and T_H_17 cells

**Figure 8:**
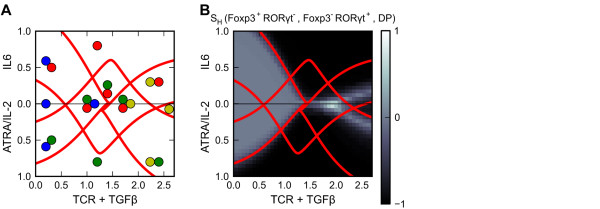
**Analyses of Prototype Model 3 (heterogeneous differentiation of iT**_**Reg**_**and T**_**H**_**17 cells). a.** Bidirectional two-parameter bifurcation diagram with respect to primary signal TCR + TGF-β and polarizing signals ATRA/IL2 and IL-6. Adjoined circles: multistability. Blue circle: naïve phenotype. Green circle: Foxp3 single-positive phenotype. Red circle: RORγt single-positive phenotype. Yellow: DP phenotype. **b.** Simulation results of induced differentiation. The heterogeneity scores with respect to Foxp3 single-positive phenotype, RORγt single-positive phenotype and DP phenotype are shown.

Heterogeneous differentiation of iT_Reg_ and T_H_17 cells has been observed in many experiments [15,16,19]. Here we present a prototype model based on the influence diagram (Figure 2C) and the parameter values (Additional file 1: Table S4). The model shows that a combination of TGF-β and TCR signal can drive a heterogeneous population containing Foxp3^+^RORγt^-^, Foxp3^-^RORγt^+^ and Foxp3^+^RORγt^+^ phenotypes (Figure 8A and B, tri-stable region at TCR + TGF-β signal ≈ 1.8). Raising the strength of TGF-β + TCR signal or adding IL-6 (a T_H_17 polarizing signal) can skew the population into Foxp3^-^RORγt^+^ and Foxp3^+^RORγt^+^ phenotypes (Figure 8A and B, bistable region in the upper plot at highest level of TCR + TGF-β signal). These results are in agreement with previous experimental observations [15,16]. Predictions made from the model include: 1) an intermediate TGF-β + TCR signal (1–1.5 units) favors heterogeneous differentiation of Foxp3^+^RORγt^-^ and Foxp3^-^RORγt^+^ populations; 2) an intermediate level of TGF-β + TCR signal (1–1.5 units) with an iT_Reg_ polarizing signal produces a homogeneous Foxp3^+^RORγt^-^ population; and 3) a high level of TGF-β + TCR signal (>2 units) with an iT_Reg_ polarizing signal induces heterogeneous Foxp3^+^RORγt^-^ and Foxp3^+^RORγt^+^ populations.

Simulation results with testable predictions are summarized in Table 6.

**Table 6:** Summary of simulation results of Prototype Model 3

**Conditions of differentiation induction**	**Induced cell population**	**Evidence**
Intermediate TGF-β + TCR signal (1.5-2 units)	Heterogeneous differentiation of Foxp3^+^RORγt^-^, Foxp3^-^RORγt^+^ and Foxp3^+^RORγt^+^ cells	[15]
High TGF-β + TCR signal (2.5 units)	Heterogeneous differentiation of Foxp3^-^RORγt^+^ and Foxp3^+^RORγt^+^ cells	[16]
Low-Intermediate TGF-β + TCR signal (1–2 units) and IL-6 signal	Heterogeneous differentiation of Foxp3^-^RORγt^+^ and Foxp3^+^RORγt^+^ cells	[15]
Low TGF-β + TCR signal (1–1.5 units)	Heterogeneous differentiation of Foxp3^+^RORγt^-^, Foxp3^-^RORγt^+^ cells	Prediction
Low-intermediate level of TGF-β + TCR signal (1–2 units) and IL-2 or ATRA	Homogeneous differentiation of Foxp3^+^RORγt^-^ cells	Prediction
High TGF-β + TCR signal (2.5 units) and IL-2 or ATRA	Heterogeneous differentiation of Foxp3^+^RORγt^-^ and Foxp3^+^RORγt^+^ cells	Prediction

## Conclusions

In this study, we have demonstrated that a simple signaling network motif can be responsible for generating all possible types of heterogeneous populations with respect to a pair of master regulators controlling CD4^+^ T cell differentiation. We showed how naïve CD4^+^ T cells can integrate multiple types of signals to differentiate into populations of diverse phenotypes. We illustrate the theoretical framework with three specific cases and made testable predictions.

It is becoming evident that certain signals can drive the differentiation of multiple lineages of T cells, whereas other environmental cues can skew the outcome to specific phenotypes [60]. Because the proposed basal motif appears commonly in the signaling networks controlling CD4^+^ T cell differentiation, biological examples of this framework are clearly not limited to the prototype models we presented here. For example, it has been recently demonstrated that STAT3 activation is required for T_H_2 differentiation [61]. This gives the possibility that IL-6, which upregulates RORγt via STAT3 activation [62], can act as a primary signal giving rise to heterogeneous T_H_2 and T_H_17 populations if the cells are primed with certain amount of other signals, such as TCR, TGFβ and IL-4.

Our study suggests the importance of regulated cell-to-cell variations that can be exploited to generate phenotypic diversity in CD4^+^ T cells. The significance of such variations in some other biological systems has been highlighted by other groups. Feinerman et al. [63] discovered that the cell-to-cell variations in the expression levels of some key co-receptors in CD8^+^ T cells can be critical for achieving diversity in TCR responses. Similarly, Chang et al. [64] demonstrated that variations in the expression of stem cell markers can influence the fate of the cell. We have used a simple generic form to account for cell-to-cell variability in this study (i.e. parametric variations), it would be interesting to study which specific variable factors in naïve CD4^+^ T cells can be predictive of the phenotypic compositions in an induced population. Harnessing such factors might be useful for fine-tuning the immune system to prevent and treat diseases.

Our modeling approach has the advantage of describing non-linear responses in biochemical reactions without knowing detailed biochemical mechanisms and kinetics, which are generally unavailable for T cell differentiation. It has the disadvantage that parameters in the equations are phenomenological and cannot be related to biochemical reaction rate constants. We expect that other modeling approaches, such as ordinary differential equations with Hill function nonlinearities, will produce results similar to ours.

We are aware of the following limitations of this framework. First, all master regulators of CD4^+^ T cell may influence each other during differentiation. Thus considering only a pair of master regulators may not be sufficient to describe all important components governing the heterogeneous differentiation of CD4^+^ T cells. Secondly, cell-to-cell communication is neglected in our models of cell population. We assume that our models describe the initial phase of differentiation and that the phenotypic compositions of the population do not change significantly during the differentiation process. The validity of this assumption needs to be examined in future studies.

## Methods

### Dynamical model

We modeled the signaling network motifs with a generic form of ordinary differential equations (ODEs) that describe both gene expression and protein interaction networks [65-67]. Each ODE in our model has the form:

(1) dXidt=γiFσiWi−XiFσW=11+e(−σW)Wi=ωio+∑jNωj→iXj,i=1,…,N

Where *X*_*i*_ is the activity or concentration of protein *i*. On a time scale = 1/γ_*i*_, *X*_*i*_(*t*) relaxes toward a value determined by the sigmoidal function, *F*, which has a steepness set by $\sigma_{i}$. The basal value of *F*, in the absence of any influencing factors, is determined by $\omega_{i}^{o}$. The coefficients $\omega_{j\to i}$ determine the influence of protein *j* on protein *i*. *N* is the total number of proteins in the network.

All variables and parameters are dimensionless. One time unit in our simulations corresponds to 1.5 days. Parameter values are listed in supplementary tables.

All simulations and bifurcation analyses were performed with PyDSTool, a software environment for dynamical systems [68].

### Bifurcation diagrams

In order to visualize the response of the T cell differentiation network to multiple signals (a primary differentiation signal and two types of polarizing signals), we have employed bidirectional two-parameter bifurcation diagrams, as in [69]. The two two-parameter bifurcation diagrams share the same primary bifurcation parameter (the primary differentiation signal, S1) on the horizontal axis. The secondary bifurcation parameters (the polarizing signals, S2 and S3) are plotted on the vertical axis: one in the upward direction and the other in the downward direction. The bidirectional two-parameter bifurcation diagram allows one to analyze the response of the regulatory system to the primary signal alone or in combination with either of the polarizing signals. Although this two-dimensional representation does not allow a full analysis of the responses to all three types of signals simultaneously, it is very useful in understanding the complex interplay between signals and responses in these heterogeneous differentiation systems. We ran simulations for a population of naïve CD4^+^ T cells, and we overlaid the simulation results on the bidirectional two-parameter bifurcation diagrams, allowing one to visualize the bifurcation analyses and simulation results simultaneously (detailed below).

### Cell-to-cell variability

To account for cell-to-cell variability in a population, we made many simulations of the system of ODEs, each time with a slightly different choice of parameter values, to represent slight differences from cell to cell. We allowed all of the parameters in our model to change simultaneously, and we assumed that the value of each parameter conforms to a normal distribution with CV = 0.05 (CV = coefficient of variation = standard deviation / mean). The mean value that we specified for each parameter distribution is also referred as the ‘basal’ value of that parameter. In our bifurcation analysis of the dynamical system, we considered an imaginary cell that adopts the basal value for each of its parameters, and we defined this cell as the ‘average’ cell. Note that none of the cells in our simulated population is likely to be this average cell, because every parameter value is likely to deviate a little (CV = 5 %) from the basal value.

In order to simulate the induced differentiation process, we first solved the ODEs numerically with some small initial values of master regulator concentrations in the absence of any exogenous signals. After a short period of time, each simulated cell will find its own, stable ‘double-negative’ steady state, corresponding to a naïve CD4^+^ T cell. Next, we changed the primary and/or polarizing signals to certain positive values and continued the numerical simulation. If needed, we continued the simulation again with a second change of primary and/or polarizing signals. By the end of the simulation, each cell arrives at its corresponding ‘induced’ phenotype, which might vary from cell to cell because of the parametric variability of the population. We repeated this simulation 200 times for a given set of exogenous signals to represent the responses of 200 cells in a population. We made the simple definition that a protein is expressed when its level is greater than 0.5 units. The simulations for a cell population were repeated 40x40 times with primary and polarizing signals of various strengths, and we overlaid the final steady state phenotypic composition on the point with corresponding coordinates on the bidirectional two-parameter bifurcation diagram.

### Mutant simulation

The experiment of knocking out GATA3-IL-4 feedback was simulated with reduced weight of auto-activation of GATA-3 to one-tenth of the original value. The experiment of knocking out T-bet genes was simulated by setting $\omega_{\text{T-bet}}^{\text{o}}$= −17 (10 times its value in the basal model).

### Heterogeneity score

To summarize simulations results with multiple phenotypes and to highlight heterogeneous and homogeneous populations in parameter space, we compute a ‘heterogeneity score’ for a simulation as follows.

(2) $$S_{\text{H}}\left(P_{1}\text{,}\ldots ,P_{n}\right)=\frac{\sum_{i=1}^{n-1}\sum_{j=i+1}^{n}\left(C_{P_{i}}+C_{P_{j}}-2\left|C_{P_{i}}-C_{P_{j}}\right|\right)}{\left(n-1\right)N}$$

The scoring function takes a list of ‘phenotypes of interest’ ($P_{1}\text{,}\ldots ,P_{n}$), and computes the sum of the pairwise heterogeneities, which are based on the numbers of cells of any two different phenotypes ($C_{P_{i}}$ and $C_{P_{j}}$). The score is normalized with respect to the number of phenotypes of interest (*n*) and the total number of cells in the population (*N*). *S*_H_ ≈ 1 when there are comparable numbers of cells of the phenotypes of interest in the population, *S*_H_ ≈ −1 when the population is dominated by one phenotype out of all the phenotypes of interest, and *S*_H_ ≈ 0 when there are few cells with the phenotypes of interest in the population, or the degree of heterogeneity is moderate.

## Competing interests

The authors declare that they have no competing interests.

## Authors’ contributions

Conceived and designed the experiments: TH JX LL JJT. Performed the experiments: TH. Analyzed the data: TH JX LL JJT. Wrote the paper: TH LL JJT. All authors read and approved the final manuscript.

## Supplementary Material

Additional file 1 4 supplementary tables and legends for supplementary figures. Click here for file

Additional file 2 **Figure S1.** Effects of primary signal saturation. Click here for file

Additional file 3 **Figure S2.** Hysteresis effect of the ‘reprogramming’ bistable switch. Click here for file

Additional file 4 **Figure S3.** Simulation results for the core motif with symmetrical parameters. Click here for file

Additional file 5 **Figure S4.** Simulation results with different relaxation rates of X and Y. Click here for file

Additional file 6 **Figure S5.** Additional bifurcation analyses of the full basal motif. Click here for file

Additional file 7 **Figure S6.** Simulation results of Prototype Model 2 (heterogeneous differentiation of T_H_1 and T_H_17 cells) with T-bet knocked-out. Click here for file
