# Supplementary material for: A simple theoretical framework for understanding heterogeneous differentiation of CD4+ T cells
Source: BMC Syst Biol. 2012 Jun 14;6:66. doi: 10.1186/1752-0509-6-66 (PMC3436737; doi:10.1186/1752-0509-6-66)
Supplement: Additional file 1 — 4 supplementary tables and legends for supplementary figures. [file 1752-0509-6-66-S1.doc]

**Supplementary Tables**

Table S 1 Parameter values for an average cell in three different generic models

| Parameter name | Description | Generic Model 1 | Generic Model 2 | Generic Model 3 |
| --- | --- | --- | --- | --- |
| $\gamma_{\text{X}}$ | Relaxation rate of X | 5 | 5 | 5 |
| $\gamma_{\text{Y}}$ | Relaxation rate of Y | 5 | 5 | 5 |
| $\sigma_{\text{X}}$ | Steepness of sigmoidal function for X | 5 | 5 | 2 |
| $\sigma_{\text{Y}}$ | Steepness of sigmoidal function for Y | 5 | 5 | 2 |
| $\omega_{\text{X} }^{\text{o}}$ | Basal activation state of X | -0.8 | -0.6 | -2.4 |
| $\omega_{\text{Y}}^{\text{o}}$ | Basal activation state of Y | -0.8 | -1 | -2.4 |
| $\omega_{\text{X}\to\text{X}}$ | Weight of autoactivation of X | 0 | 0 | 3.2 |
| $\omega_{\text{Y}\to\text{X}}$ | Weight of inhibition on X by Y | -1.5 | -2.5 | -1 |
| $\omega_{\text{Y}\to\text{Y}}$ | Weight of autoactivation of Y | 0 | 0 | 3.2 |
| $\omega_{\text{X} \to\text{Y}}$ | Weight of inhibition on Y by X | -1.5 | -1 | -1 |
| $\omega_{\text{S}1\to\text{X} }$ | Weight of activation on X by S1 | 1 | 1 | 1 |
| $\omega_{\text{S}1\to\text{Y}}$ | Weight of activation on Y by S1 | 1 | 0.8 | 1 |
| $\omega_{\text{S}2\to\text{X}}$ | Weight of activation on X by S2 | 1 | 1 | 1 |
| $\omega_{\text{S}3\to\text{Y}}$ | Weight of activation on Y by S3 | 1 | 1 | 1 |
| S1 | Strength of primary signal S1 | 0 - 3* | 0 - 3 | 0 - 2.5 |
| S2 | Strength of polarizing signal S2 | 0 - 0.4 | 0 - 0.6 | 0 - 1.3 |
| S3 | Strength of polarizing signal S3 | 0 - 0.4 | 0 - 0.6 | 0 - 1.3 |

* S1 is replaced by sigmoidal function $1.5/(1+e^{2\cdot(-1+\text{S1})})$in Figure S 1A and B to illustrate the effect of primary signal saturation.

Table S 2 Parameter values for an average cell in Prototype Model 1 (T_H_1-T_H_2)

| Parameter name | Description | Value |
| --- | --- | --- |
| $\gamma_{\text{T-bet}}$ | Relaxation rate of T-bet | 5 |
| $\gamma_{\text{GATA3}}$ | Relaxation rate of GATA3 | 5 |
| $\sigma_{\text{T-bet}}$ | Steepness of sigmoidal function for T-bet | 4 |
| $\sigma_{\text{GATA3}}$ | Steepness of sigmoidal function for GATA3 | 6 |
| $\omega_{\text{T-bet} }^{\text{o}}$ | Basal activation state of T-bet | -1.7 |
| $\omega_{\text{GATA3}}^{\text{o}}$ | Basal activation state of GATA3 | -2 |
| $\omega_{\text{T-bet}\to\text{T-bet}}$ | Weight of autoactivation of T-bet | 2 |
| $\omega_{\text{GATA3}\to\text{T-bet}}$ | Weight of inhibition on T-bet by GATA3 | -2 |
| $\omega_{\text{GATA3}\to\text{GATA3}}$ | Weight of autoactivation of GATA3 | 2.5 (0.25) |
| $\omega_{\text{T-bet} \to\text{GATA3}}$ | Weight of inhibition on GATA3 by T-bet | -1 |
| $\omega_{\text{TCR}\to\text{T-bet} }$ | Weight of activation on T-bet by TCR | 1 |
| $\omega_{\text{TCR}\to\text{GATA3}}$ | Weight of activation on GATA3 by TCR | 1 |
| $\omega_{\text{IL}-12\to\text{T-bet}}$ | Weight of activation on T-bet by IL-12 | 1 |
| $\omega_{\text{IL}-4\to\text{GATA3}}$ | Weight of activation on GATA3 by IL-4 | 1 |
| TCR | Strength of TCR signal | 0 - 2.3 |
| IL-12 | Strength of IL-12 signal | 0 - 0.7 |
| IL-4 | Strength of IL-4 signal | 0 - 1.2 |

Table S 3 Parameter values for an average cell in Prototype Model 2 (T_H_1-T_H_17)

| Parameter name | Description | Value |
| --- | --- | --- |
| $\gamma_{\text{T-bet}}$ | Relaxation rate of T-bet | 5 |
| $\gamma_{\text{RORγ} \text{t}}$ | Relaxation rate of RORγt | 5 |
| $\sigma_{\text{T-bet}}$ | Steepness of sigmoidal function for T-bet | 4 |
| $\sigma_{\text{RORγ} \text{t}}$ | Steepness of sigmoidal function for RORγt | 3 |
| $\omega_{\text{T-bet} }^{\text{o}}$ | Basal activation state of T-bet | -1.7 |
| $\omega_{\text{RORγ} \text{t}}^{\text{o}}$ | Basal activation state of RORγt | -2.7 |
| $\omega_{\text{T-bet}\to\text{T-bet}}$ | Weight of autoactivation of T-bet | 2 |
| $\omega_{\text{RORγ} \text{t}\to\text{T-bet}}$ | Weight of inhibition on T-bet by RORγt | -1 |
| $\omega_{\text{RORγ} \text{t}\to\text{RORγ} \text{t}}$ | Weight of autoactivation of RORγt | 2 |
| $\omega_{\text{T-bet} \to\text{RORγ} \text{t}}$ | Weight of inhibition on RORγt by T-bet | -0.8 |
| $\omega_{\text{TCR}\to\text{T-bet} }$ | Weight of activation on T-bet by TCR | 2.2 |
| $\omega_{\text{TCR}\to\text{RORγ} \text{t}}$ | Weight of activation on RORγt by TCR | 2.2 |
| $\omega_{\text{IL23}+\text{IL1}\to\text{RORγ} \text{t}}$ | Weight of activation on RORγt by IL-23+IL-1 | 1 |
| $\omega_{\text{TGFβ}+\text{IL6}\to\text{RORγ} \text{t}}$ | Weight of activation on RORγt by TGFβ+IL-6 | 1 |
| $\omega_{\text{TGFβ}+\text{IL6}\to\text{T-bet}}$ | Weight of inhibition on T-bet by TGFβ+IL-6 | -1 |
| TCR | Strength of TCR signal | $1/(1+e^{3\cdot(-1+0.8\cdot\text{S1})})$ $0\leq\text{S1}\leq3$ |
| IL23+IL1 | Strength of IL-23+IL-1 signal | 0 - 1 |
| TGFβ+IL6 | Strength of TGFβ+IL-6 signal | 0 - 1 |

Table S 4 Parameter values for an average cell in Prototype Model 3 (iT_Reg_-T_H_17)

| Parameter name | Description | Value |
| --- | --- | --- |
| $\gamma_{\text{Foxp3}}$ | Relaxation rate of Foxp3 | 5 |
| $\gamma_{\text{RORγ} \text{t}}$ | Relaxation rate of RORγt | 5 |
| $\sigma_{\text{Foxp3}}$ | Steepness of sigmoidal function for Foxp3 | 2.8 |
| $\sigma_{\text{RORγ} \text{t}}$ | Steepness of sigmoidal function for RORγt | 3 |
| $\omega_{\text{Foxp3} }^{\text{o}}$ | Basal activation state of Foxp3 | -2.5 |
| $\omega_{\text{RORγ} \text{t}}^{\text{o}}$ | Basal activation state of RORγt | -2.5 |
| $\omega_{\text{Foxp3}\to\text{Foxp3}}$ | Weight of autoactivation of Foxp3 | 3 |
| $\omega_{\text{RORγ} \text{t}\to\text{Foxp3}}$ | Weight of inhibition on Foxp3 by RORγt | -1 |
| $\omega_{\text{RORγ} \text{t}\to\text{RORγ} \text{t}}$ | Weight of autoactivation of RORγt | 3 |
| $\omega_{\text{Foxp3}\to\text{RORγ} \text{t}}$ | Weight of inhibition on RORγt by Foxp3 | -0.8 |
| $\omega_{\text{TCR}+\text{TGFβ}\to\text{Foxp3} }$ | Weight of activation on Foxp3 by TCR+TGF-β | 2.6 |
| $\omega_{\text{TCR}+\text{TGFβ}\to\text{RORγ} \text{t}}$ | Weight of activation on RORγt by TCR+TGF-β | 2.6 |
| $\omega_{\text{ATRA/IL2}\to\text{Foxp3}}$ | Weight of activation on Foxp3 by ATRA/IL-2 | 1.5 |
| $\omega_{\text{IL6}\to\text{RORγ} \text{t}}$ | Weight of activation on RORγt by IL-6 | 1 |
| $\omega_{\text{ATRA/IL2}\to\text{RORγ} \text{t} }$ | Weight of inhibition on Foxp3 by ATRA/IL-2 | -1 |
| $\omega_{\text{IL6}\to\text{Foxp3}}$ | Weight of inhibition on Foxp3 by IL-6 | -1.5 |
| TCR+TGFβ | Strength of TCR+TGFβ signal | $1/(1+e^{2.5\cdot(-1+0.8\cdot\text{S1})})$ $0\leq\text{S1}\leq2.6$ |
| ATRA/IL2 | Strength of ATRA/IL-2 signal | 0 - 1 |
| IL6 | Strength of IL-6 signal | 0 - 1 |

**Legends for supplementary figures**

Figure S 1. Effects of primary signal saturation. A. One-parameter bifurcation diagram for steady state level of X as a function of primary signal S1, which saturates at 1.5 units. Solid curve: stable steady state; dashed curve: unstable steady state. B. Bidirectional two-parameter bifurcation diagram with respect to primary signal S1 and polarizing signals S2 and S3. S1 saturates at 1.5 units. Solid curve: locus of bifurcation points in Panel A. The types of stable steady states are annotated as colored circles. Adjoined circles: multistability. See Figure 1 for interpretation of the color scheme.

Figure S 2. Hysteresis effect of the ‘reprogramming’ bistable switch. A. One-parameter bifurcation diagram for steady state level of X as a function of primary signal S1 in the presence of polarizing signal S2. B. One-parameter bifurcation diagram for steady state level of Y as a function of primary signal S1 in the presence of polarizing signal S2. C. One-parameter bifurcation diagram for steady state level of X as a function of polarizing signals S2 and S3 in the presence of primary signal S1=1.5. D. One-parameter bifurcation diagram for steady state level of Y as a function of polarizing signals S2 and S3 in the presence of primary signal S1=1.5. E. Bidirectional two-parameter bifurcation diagram with respect to primary signal S1 and polarizing signals S2 and S3. Horizontal line: reference to diagram shown in Panels A and B. Vertical line: reference to diagrams shown in Panels C and D. The types of stable steady states are annotated as colored circles. Adjoined circles: multistability. See Figure 1 for interpretation of the color scheme.

Figure S 3. Simulation results for the core motif with symmetrical parameters. A. Percentages of naïve cells at the end of the simulations plotted on the bidirectional two-parameter bifurcation diagram. B. Percentages of XSP cells at the end of the simulations plotted on the bidirectional two-parameter bifurcation diagram. C. Percentages of YSP cells at the end of the simulations plotted on the bidirectional two-parameter bifurcation diagram. D. Percentages of DP cells at the end of the simulations plotted on the bidirectional two-parameter bifurcation diagram (compare Panels A-D with Figure 3G). E. Overlaid phase plane portraits. The small cell-to-cell variability make the naïve states (magenta circles) of individual cells lie on different sides of the separatrices (gray curves), thereby resulting different fates of the cells. Green curve: X nullcline. Red curve: Y nullcline. Closed circle: stable steady state. Open circle: unstable steady state.

**Figure S 4. Simulation results with different relaxation rates of X and Y. A.** Simulation results for the core motif with symmetrical parameters (compare with Figure 3G). **B.** Simulation results for the core motif with asymmetrical parameters (compare with Figure 4D). In each panel, heterogeneity scores with respect to XSP and YSP are shown. In both simulations, the relaxation rate of Y ($\gamma_{\text{Y}}$) is changed to 10 (twice of the rate of X).

Figure S 5. Additional bifurcation analyses of the full basal motif. A. One-parameter bifurcation diagram for steady state level of X as a function of primary signal S1 (S2=S3=0) for the case of intermediate weights (=1.5) of auto-activation relations. B. One-parameter bifurcation diagram for steady state level of X as a function of primary signal S1 (S2=S3=0) for higher weights (=3.2) of auto-activation relations. C. Two-parameter bifurcation diagram with respect to the weights of auto-activation relations and the primary signal S1 (S2=S3=0). Purple curve: locus of the supercritical pitchfork bifurcation points. Orange curve: locus of the subcritical bifurcation points. Red curve: locus of the saddle-node bifurcation points. Gray lines: reference to diagrams shown in Panels A and B. D. One-parameter bifurcation diagram for steady state level of X as a function of primary signal S1 in the presence of polarizing signal S2. E. One-parameter bifurcation diagram for steady state level of Y as a function of primary signal S1 in the presence of polarizing signal S2. F. Bidirectional two-parameter bifurcation diagram with respect to primary signal S1 and polarizing signals S2 and S3. Gray lines: references to diagram shown in Panels D and E. In Panels A, B, D and E: Sold curve: stable steady state; dashed curve: unstable steady state. In Panels C and F, the types of stable steady states are annotated as colored circles. Adjoined circles: multistability. See Figure 1 for interpretation of the color scheme.

Figure S 6. Simulation results of Prototype Model 2 (heterogeneous differentiation of T_H_1 and T_H_17 cells) with T-bet knocked-out. A. Two-parameter bifurcation diagram with respect to primary signal TCR and polarizing signal IL-23 + IL1. Adjoined circles: multistability. Blue circle: naïve phenotype. Green circle: T-bet single-positive phenotype. Red circle: RORγt single positive phenotype. Yellow: DP phenotype. B. Simulation results for induced differentiation. Heterogeneity scores with respect to T-bet single-positive phenotype and RORγt single-positive phenotype are shown. (Bifurcation diagram and simulation results with respect to primary signal TCR and polarizing signal TGF-β + IL-6 are identical to Panels A and B.)
