# Supplementary figures and images for: A simple theoretical framework for understanding heterogeneous differentiation of CD4+ T cells
Source: BMC Syst Biol. 2012 Jun 14;6:66. doi: 10.1186/1752-0509-6-66 (PMC3436737; doi:10.1186/1752-0509-6-66)

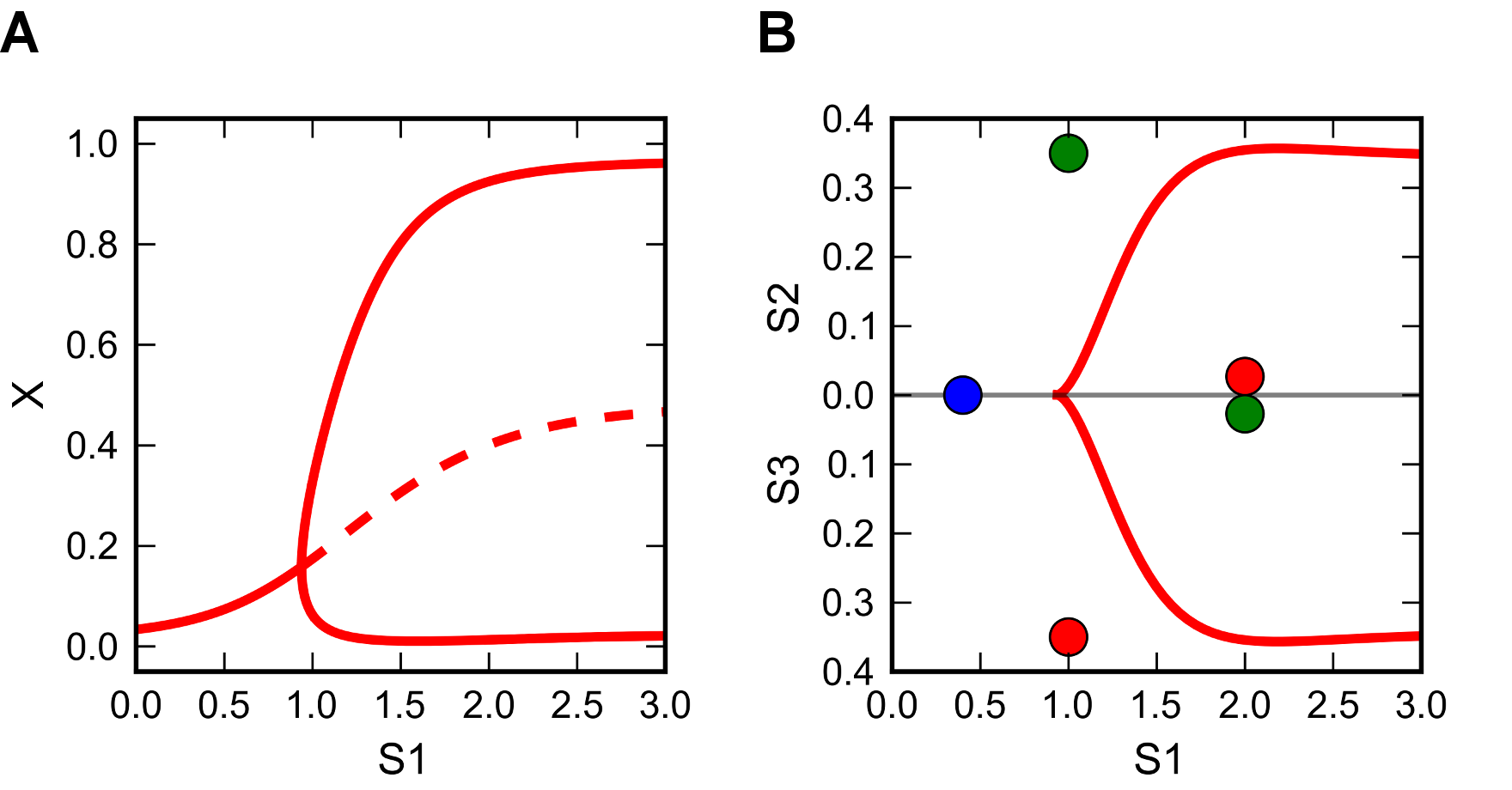

Supplement: Additional file 2 — Figure S1. Effects of primary signal saturation. [file 1752-0509-6-66-S2.tiff]

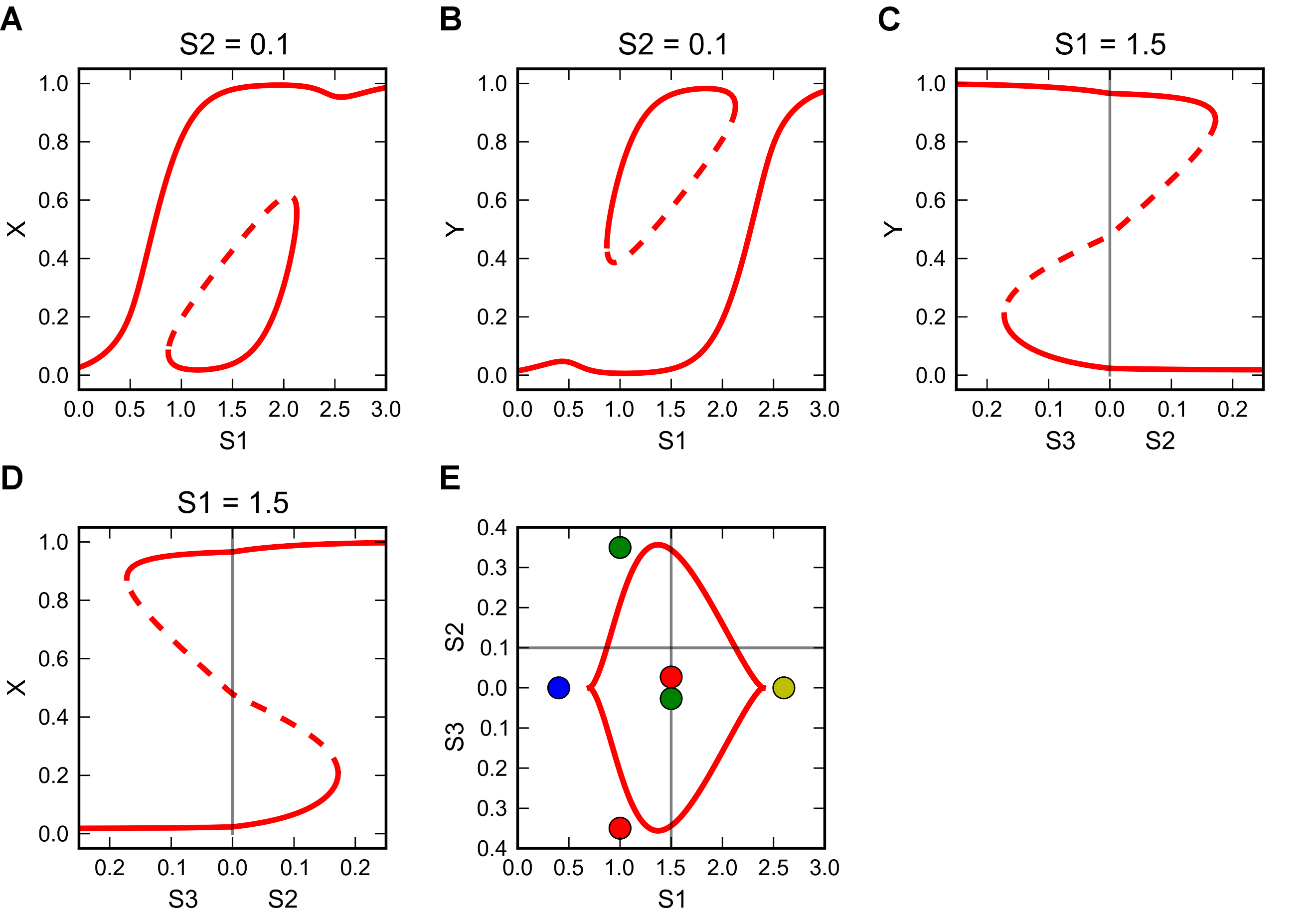

Supplement: Additional file 3 — Figure S2. Hysteresis effect of the ‘reprogramming’ bistable switch. [file 1752-0509-6-66-S3.tiff]

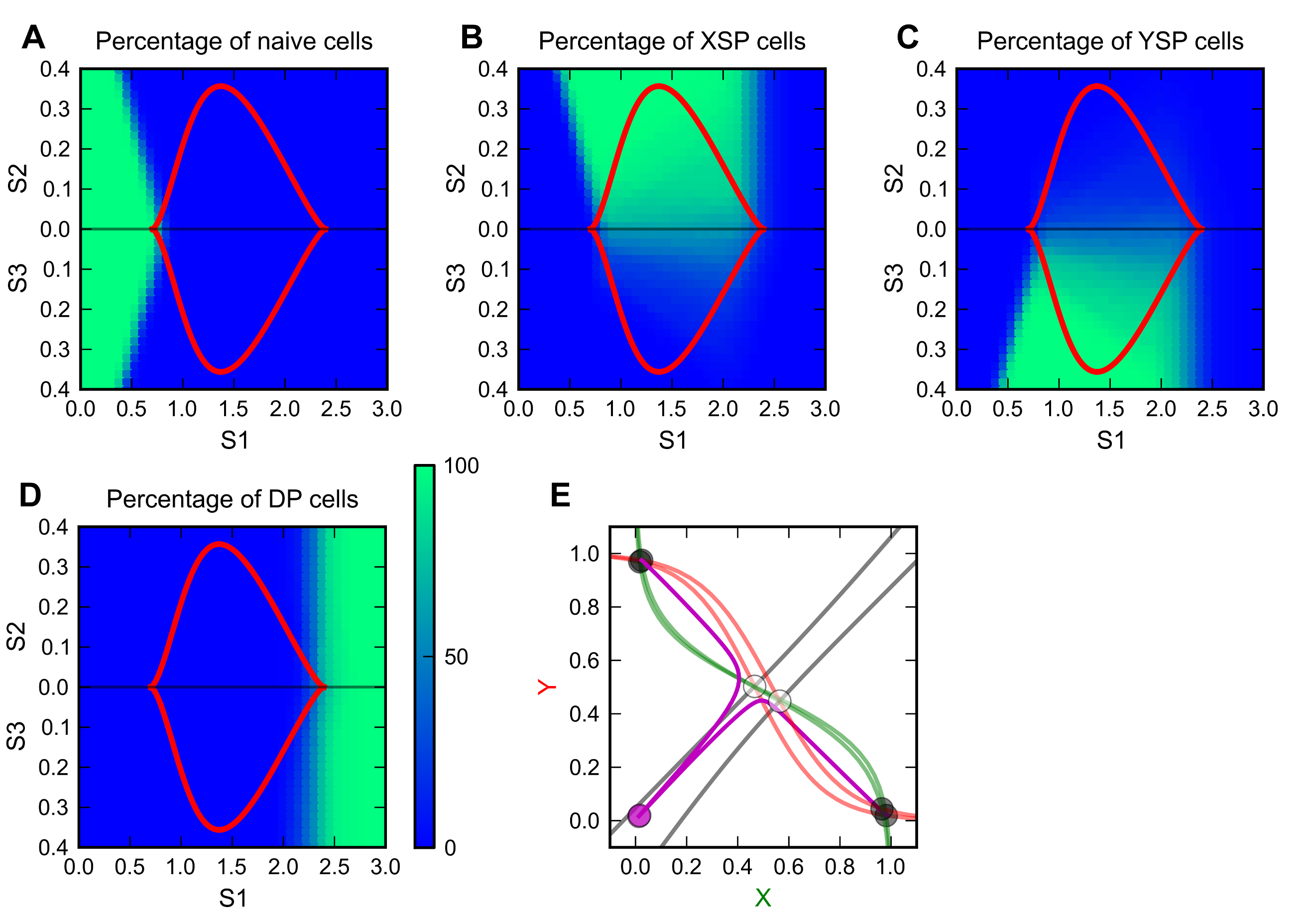

Supplement: Additional file 4 — Figure S3. Simulation results for the core motif with symmetrical parameters. [file 1752-0509-6-66-S4.tiff]

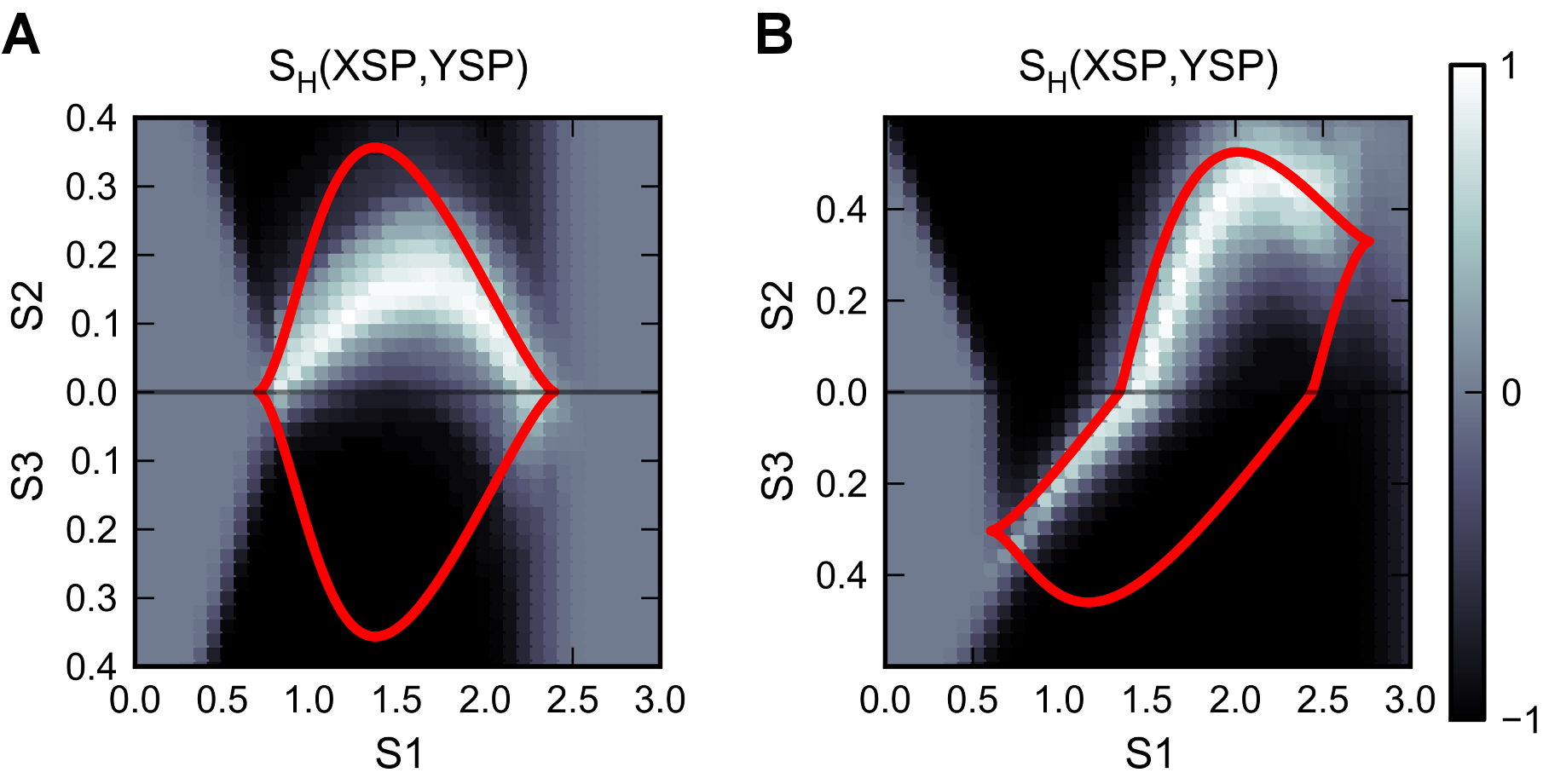

Supplement: Additional file 5 — Figure S4. Simulation results with different relaxation rates of X and Y. [file 1752-0509-6-66-S5.tiff]

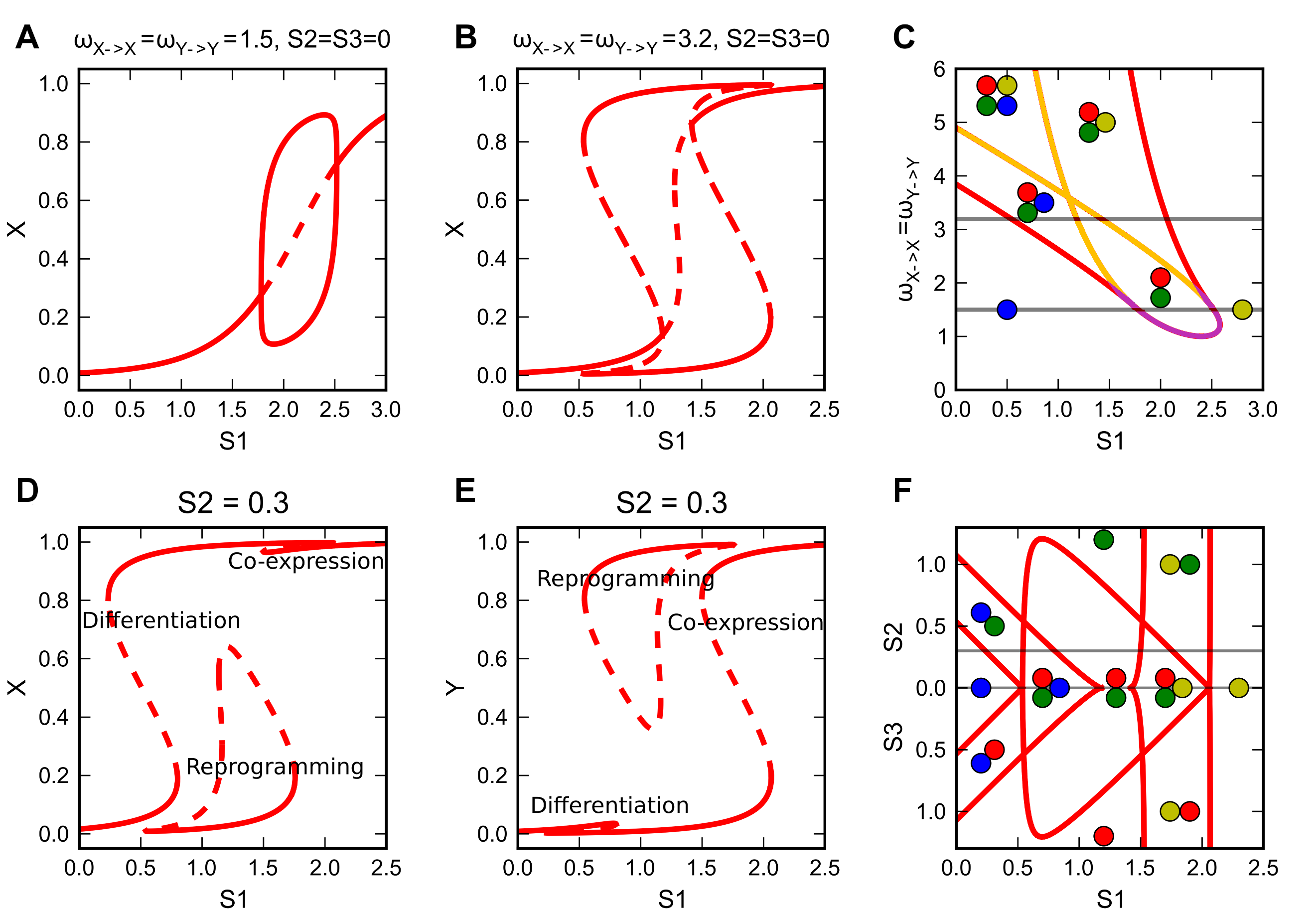

Supplement: Additional file 6 — Figure S5. Additional bifurcation analyses of the full basal motif. [file 1752-0509-6-66-S6.tiff]

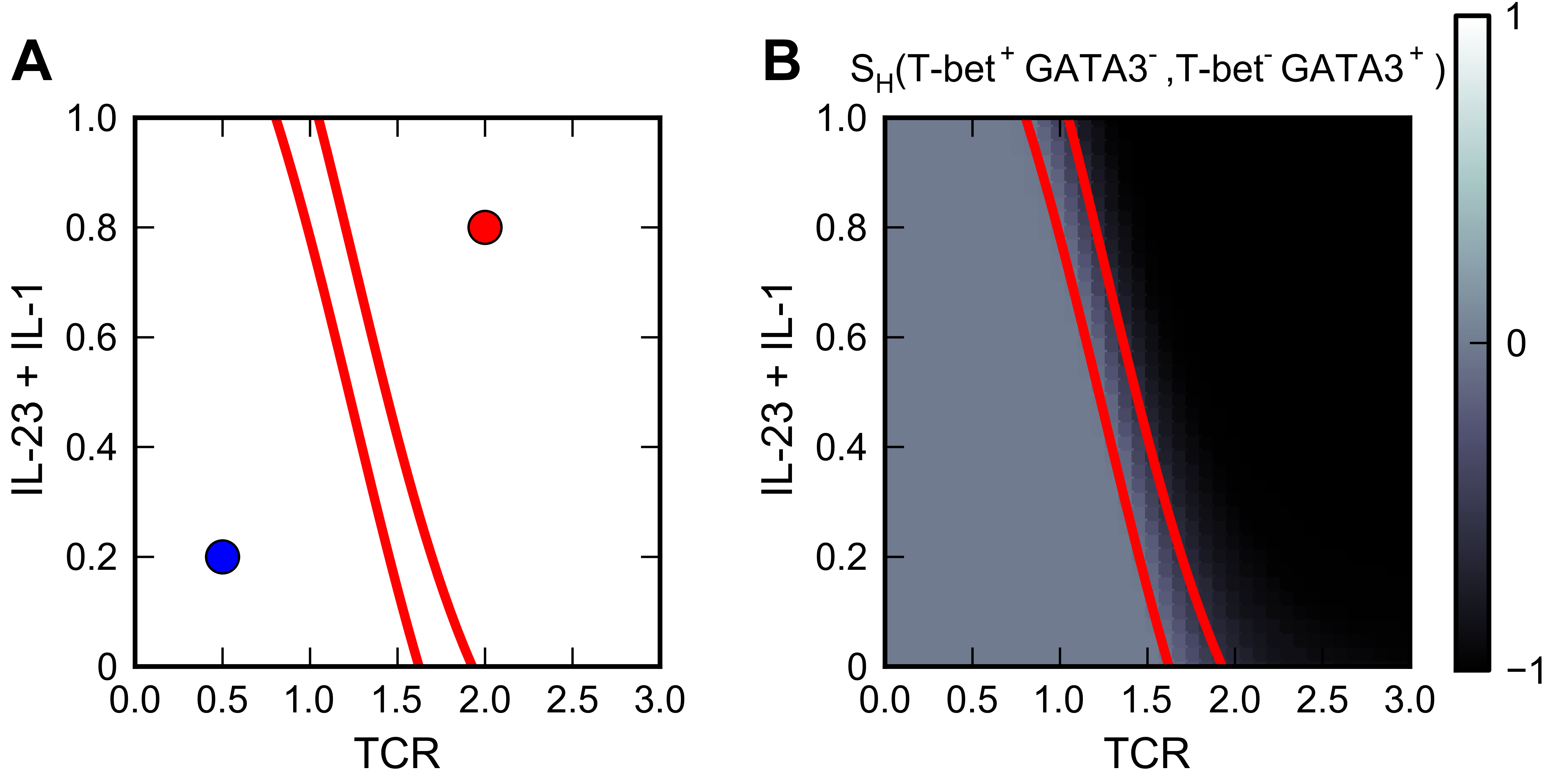

Supplement: Additional file 7 — Figure S6. Simulation results of Prototype Model 2 (heterogeneous differentiation of TH1 and TH17 cells) with T-bet knocked-out. [file 1752-0509-6-66-S7.tiff]
